# The *in vitro* Analysis of Quality of Ovarian Follicle Culture Systems Using Time-Lapse Microscopy and Quantitative Real-Time PCR

**Published:** 2020

**Authors:** Maxim Alexeevich Filatov, Denis Alexandrovich Nikishin, Yulia Vladimirovna Khramova, Maria L’vovna Semenova

**Affiliations:** 1. Faculty of Biology, Lomonosov Moscow State University, Moscow, Russia; 2. N.K. Koltzov Institute of Developmental Biology, Russian Academy of Sciences, Moscow, Russia

**Keywords:** Alginate, *In vitro* culture, Mice, Oocyte, Ovarian follicle, Real-time PCR, Time-lapse microscopy

## Abstract

**Background:**

The aim of ovarian follicle *in vitro* culture is to obtain mature oocytes. To evaluate the efficiency of *in vitro* culture system, the status of the cultured oocyte can be analyzed.

**Methods:**

The preantral ovarian follicles retrieved from 14-day-old C57Bl/6J mice were cultured in 3D alginate hydrogel. The status of oocytes obtained from mature (3 months old, group A) and immature (3 weeks old, group B) mice was compared to the status of oocytes retrieved from ovarian follicles cultured *in vitro* (Group C) using qRT-PCR analysis and time-lapse microscopy. In the qRT-PCR analysis, 8 samples for group A (80 oocytes), 8 samples for group B (80 oocytes), and 6 samples for group C (60 oocytes) were included. Time-lapse analysis was performed in group A (oocytes n=31), group B (n=45), and group C (n=21). Statistical analysis was done by Kruskal-Wallis and chi-square tests and differences were considered statistically significant if p<0,05.

**Results:**

The diameter of group C oocytes is lower in comparison to group A oocytes (67 *μm vs*. 75 *μm*, correspondingly). Groups B and C oocytes exhibited delayed meiosis in comparison to group A oocytes. Expression levels of six oocyte maturation genes (Ccnb, CDK1, Ccnh, Wee2, Mos and Epab) were evaluated using qRT-PCR analysis. Expression levels of Ccnh and Epab are lowered in group C oocytes compared to the expression levels of these genes in groups A and B oocytes (p< 0.05).

**Conclusion:**

Oocytes obtained after ovarian follicles *in vitro* culture have reduced development competence, future fundamental changes of *in vitro* culture systems can be expected.

## Introduction

In recent years, number of research works dedicated to the ovarian follicle *in vitro* culture has increased. *In vitro* culture of ovarian follicles appears to be one of the most potentially applicable methods of fertility preservation for women who suffer from oncological or autoimmune diseases (1). The methods of ovarian tissue culture and individual follicle *in vitro* culture are currently being actively developed. Mouse ovarian tissue is commonly used as a model for studies in the field of ovarian tissue *in vitro* culture (2–6). Traditionally 2D or 3D (Which are considered more efficient) *in vitro* culture systems of ovarian follicles are used. Basically in 3D systems, ovarian follicles are encapsulated in different hydrogels and cultured in specific culture mediums containing additives which stimulate folliculogenesis (7). Although the cases of mouse healthy pups birth after fertilization of oocytes obtained from ovarian follicles cultured *in vitro* have been reported (8–11), the regulation of follicle growth and oocyte maturation *in vitro* is not comprehensively investigated.

Quality evaluation of the ovarian follicle *in vitro* culture systems is one of the most important tasks. Commonly used criteria for quality evaluation of ovarian follicle *in vitro* culture systems is the percentage of MII oocytes (Which are presumed as mature oocytes) obtained at the end of culture (12–16). Nevertheless, if oocyte reaches MII stage it does not indicate that this oocyte is capable of fertilization and further development (17). Therefore, MII oocytes production could only be a relative indicator of applied culture protocol efficiency.

Another approach used to analyze the quality of ovarian follicle *in vitro* culture systems is analysis of gene expression levels in cultured ovarian follicles (13, 18–20). Undoubtedly, there is a cross talking between follicular cells and oocyte, and if follicular cells are not “healthy” then oocyte probably will not be able for normal development. However, it should be considered that generally when gene expression levels analysis of ovarian follicles is performed, only few genes are analyzed, and these genes may show normal expression levels and the other genes may show abnormal expression pattern. Furthermore, even when gene expression levels are normal, protein synthesis may be disturbed and cross talking between oocyte and follicular cells may be broken. Thus, analysis of gene expression levels in ovarian follicles cultured *in vitro* may show that ovarian follicle status is good while the competence of obtained oocytes is insufficient.

The principle goal of studies dedicated to the ovarian follicle *in vitro* culture systems is to obtain mature oocytes which are capable of fertilization and further development (7). Therefore, it seems that the most adequate approach to evaluate the efficiency of ovarian follicle *in vitro* culture system is to analyze directly the status of the oocyte. There are only a few research works devoted to the investigation of gene expression levels in oocytes obtained from ovarian follicles cultured *in vitro* (4, 21). In the work of Sánchez et al. (21), expression levels of Gdf9, Mater, Nmp2 in oocytes were measured. In the work of Jiao and Woodruff (4), relative transcript levels of Gdf9, Bmp15, Nlrp5, Tcl1, and Zp3 were measured. All the genes mentioned above play important roles in oocyte development. Nevertheless, the number of genes which are essential for oocyte maturation is significantly larger. In the current work, other genes which are necessary for oocyte maturation were the center of focus: Cdk1, Ccnb, Ccnh, Wee2, Mos and Epab (17). Cdk1 and Ccnb are encoding subunits of MPF (Maturation promoting factor) complex: CDK1 (also known as CDC2 or p34Cdc2) and Cyclin B1, respectively. MPF-complex is a crucial component required for GVBD (Germinal vesicle breakdown) and meiosis initiation (17). Ccnh and Wee2 are encoding Cyclin H and WEE2 proteins, respectively. These proteins play significant roles in regulation of MPF complex activity. MOS is an important regulator of meiotic maturation which controls MAPK-pathway. MAPK-pathway performs multiple functions in oocyte meiosis: activation of MAPK-pathway leads to GVBD (22–23); MAPK-pathway is also responsible for normal spindle assembly in oocytes (24). Moreover, MAPK cascade regulates maternal mRNA translation in oocytes (25). Epab gene is responsible for synthesis of ePAB (Embryonic poly (A)-binding) protein. This protein is specific for oocytes; it is bound to the poly-A terminal sites of mRNA and triggers their translation (26–28).

Time-lapse microscopy is a commonly used technique in the area related to the early embryo development of mammalian embryos (29–30). This technique allows observing different morphological stages during cleavage and blastocyst formation (31–32). Moreover, time-lapse microscopy could be applicable to monitor morphological changes in oocytes during meiosis (33–35). Therefore, in the current work, time-lapse microscopy was also applied to investigate mechanisms of meiotic progression in oocytes obtained after *in vitro* culture of ovarian follicles.

## Methods

### Materials:

All the laboratory culture dishes used for the current study were purchased from SPL, Lifesciences unless stated otherwise. The dishes used for molecular analysis were purchased from Axygen (Corning, USA). Insulin-transferrin-selenium, phosphate buffer saline (PBS), epidermal growth factor (EGF) and Minimum Essential Medium Eagle–Alpha Modification (α-MEM) were purchased from PanEco (Russia). Fetal calf serum (FCS) was purchased from Hyclone (Thermo-Fischer, USA). Human Tubal Fluid was purchased from Irvine Scientific (USA). Gentamycin and ascorbic acid were purchased from Dalhimfarm (Russia). Human chorionic gonado-tropin (hCG) and follicle stimulating hormone (FSH) were purchased from Merck Serono (Switzerland). All other reagents were purchased from Sigma-Aldrich (USA) unless stated otherwise.

### Animals:

C57BL/6J mice were purchased from animal house of Federal Medical Biological Agency, Branch “Andreevka”. Animals were maintained under controlled conditions (22–24*°C* and 14L: 10D photoperiod). Mice were given ad libitum access to food and water. All procedures performed in studies involving animals were in accordance with the ethical standards of the Moscow State University Bioethical Committee and with the 1964 Helsinki declaration and its later amendments. Ethical approval documentation registration number is 71-j and date of registration was March 26, 2018.

### Ovarian follicles collection and in vitro culture:

**Figure 1.**
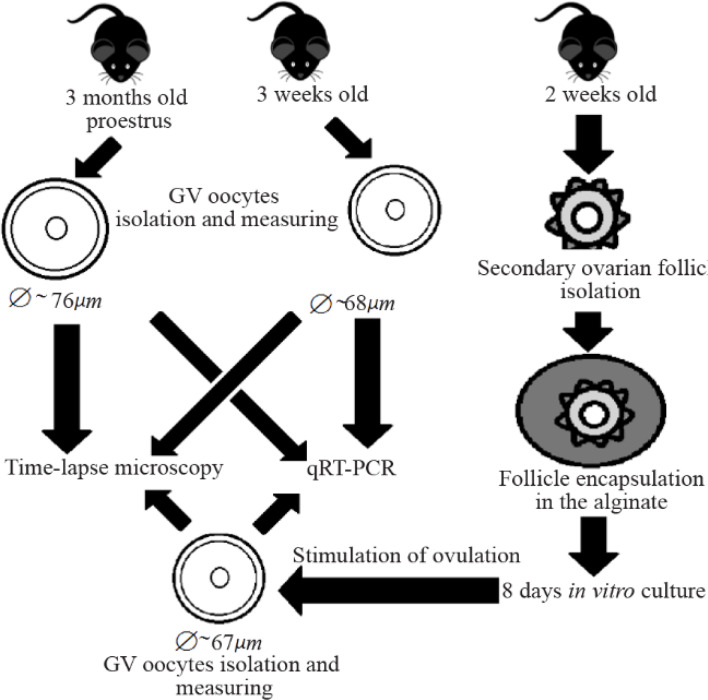
Scheme representing principle steps of work

The principle scheme illustrating the main steps of the work is represented in figure 1. In this work, multi-layered preantral ovarian follicles retrieved from 14-day-old mice were used. Mice were sacrificed by cervical dislocation, then ovaries were removed and placed into the HEPES-containing medium (Human Tubal Fluid Medium, Irvine Scientific) and pre-equilibrated in the CO_2_-incubator (5% CO_2_/95% air, 37*°C*). Then, ovaries were dissected using insulin needles and secondary ovarian follicles were collected.

Ovarian follicles were placed into the alginate solution (0.75%) diluted with PBS. Alginate solution was pre-equilibrated in the CO_2_-incubator. Then, 15 *μl* drops of alginate solution were placed for 2 *min* in 0.2 *M* solution of CaCl_2_ (Diluted with distilled water and pre-equilibrated in the CO_2_-incubator) for alginate polymerization and hydrogel formation. Each drop contained one ovarian follicle. After that, each drop with encapsulated ovarian follicle was transferred with forceps into the Petri dish with α-MEM medium for washing. Each drop was washed two times and then placed into the Start culture medium. Each alginate drop with encapsulated ovarian follicle was placed in the well of 4-well culture dish with 600 *μl* of culture medium and cultured in the CO_2_-incubator. To avoid evaporation of culture medium, 1 *ml* of α-MEM was added into the inter-well space of the plate.

Three types of culture medium were used: Start culture medium (S-medium), Prolongation culture medium (P-medium), and Maturation culture medium (M-medium). S-medium was used during the first two days of ovarian follicle culture. S-medium was based on α-MEM with addition of L-carnitine (1 *mM*), ascorbic acid (50 *μg/ml*), FSH (0.1 *IU/ml*), FCS (5%), insulin (5 *μg/ml*), transferrin (5 *μg/ml*), selenium (5 *ng/ml*), and gentamycin (80 *μg/ml*). Subsequently, each two days, the half of the medium was replaced with P-medium. P-medium was also based on α-MEM and contained L-carnitine (2 *mM*), ascorbic acid (100 *μg/ml*), FSH (0.2 *IU/ml*), FCS (10%), insulin (10 *μg/ml*), transferrin (10 *μg/ml*), selenium (10 *ng/ml*), and gentamycin (160 *μg/ml*). After eight days of culture, expansive growth of follicles was observed and half of the medium was changed to M-medium which also was based on α-MEM and contained L-carnitine (2 *mM*), hCG (3 *IU/ml*), FSH (0.2 *IU/ml*), FCS (10%), insulin (10 *μg/ml*), transferrin (10 *μg/ml*), selenium (10 *ng/ml*), gentamycin (160 *μg/ml*), EGF (10 *ng/ml*), and pimobendan (70 *μg/ml*) to prevent GV breakdown. After that, ovarian follicles were cultured for 4 hours and then oocytes were retrieved from follicles.

### Oocyte collection:

In the current work, three groups of oocytes were used. First group (group A) contained oocytes obtained from the immature mice (22 days old). The second group (group B) of oocytes was retrieved from the adult mice (3 months old) at the proestrus phase of the estrous cycle. The third group (Group C) contained oocytes retrieved from ovarian follicles cultured *in vitro* as described above.

Phases of estrous cycle were estimated using vaginal smear method as described (36). Mice were sacrificed by cervical dislocation, then ovaries were removed and placed into the HEPES-containing medium pre-equilibrated in the CO_2_-incubator. Pimobendan (A selective blocker of PDE3A) was added directly before usage into the HEPES-containing medium to get a final concentration of 35 *μg/ml* in order to prevent premature GV breakdown in obtained oocytes. Then, ovaries were dissected using insulin needles and oocytes were collected.

To obtain oocytes from follicles cultured *in vitro* the following actions were performed. Ovarian follicles were retrieved mechanically from alginate hydrogel using insulin needles, then, oocytes were retrieved from ovarian follicles mechanically using insulin needles. Oocytes were divided into groups of 10 oocytes each (One sample), washed three times with HEPES-containing medium without addition of PDE3A inhibitor and then were subjected to qRT-PCR analysis. For the qRT-PCR, 8 samples in group A, 8 samples in group B and 6 samples in group C, totally 22 samples were used (220 oocytes).

### cDNA synthesis:

Synthesis of cDNA library was carried out using the commercial kit SuperScript III cDNA Synthesis CellDirect (ThermoFisher, USA) following the manufacturer’s instructions. All obtained cDNA samples were stored at −20°*C* until qRT-PCR analysis.

### Primer design:

Primers (Table 1) were designed by Lasergene PrimerSelect (DNASTAR) software and online utility Blast ( https://www.ncbi.nlm.nih.gov/tools/primer-blast/ ) using sequences from the NCBI GenBank database.

**Table 1.** Target and reference genes selected for qRT-PCR analysis of oocytes

**Target gene**	**GenBank accession no.**	**Localization and primer sequence**	**Product size (*bp*)**	**Annealing temperature (*°C*)**
**Ccnb**	NM_172301.3	Exon 1F: 5′-TCTCGAATCGGGGAACCTC-3′	106	61.7
		Exon 2R: 5′-GCGCCTGCCATACTGACC-3′		61.6
**Ccnh**	NM_023243.5	Exon 7F: 5′-AAGCTGGAGCGGTGTCATTCT-3′	174	63.2
		Exon 9R: 5′-AAGTACTGCTGCGGTCATTTATT-3′		59.9
**Cdk1**	NM_007659.3	Exon 5F: 5′-CCCGGCGAGTTCTTCACA-3′	173	62.1
		Exon 6R: 5′-CGAGCCCAGCAACACTTCT-3′		60.6
**Epab**	NM_001114079.2	Exon 13F: 5′-CCTGCGCCCCACTGACT-3′	123	61.9
		Exon 14R: 5′-GTACTGCCACCGCCTCTTCTAT-3′		62.1
**Gapdh**	NM_001289726.1	Exon 5F: 5′-GACGTGCCGCCTGGAGA-3′	144	63.0
		Exon 6R: 5′-GAAGAGTGGGAGTTGCTGTTGAA-3′		62.9
**H2afz**	NM_016750.3	Exon 4–5F: 5′-ATTGCTGGTGGTGGTGTCATC-3′	212	62.3
		Exon 5R: 5′-GCCTCCAACTTGCTCAAATAGAAT-3′		62.9
**Mos**	NM_020021.2	Exon 1F: 5′-TGTTAACGGCCTGCTTTTTC-3′	171	60.2
		Exon 1R: 5′-GTGCCCCCTATGTGGTGAG-3′		60.3
**Rpl4**	NM_024212.4	Exon 1–2F: 5′-TCCCCGTCATGGCTTGTG-3′	169	62.7
		Exon 2R: 5′-ACGGCATAGGGCTGTCTGTT-3′		61.6
**Wee2**	NM_201370.2	Exon 10F: 5′-GAGCTCTCGGATGACTTTTATGGT-3′	186	62.7
		Exon 11–12R: 5′-TTTCAGTTCCCTTTTCAGTGTGG-3′		62.8

### Quantitative real-time PCR:

Quantitative RT-PCR was performed on a StepOnePlus real-time PCR system (Applied BioSystems, USA) using SYBR® green I with ROX as an internal loading standard. The qPCRmix-HS SYBR+ROX kit (Evrogen, Russia) was utilized for all reference gene qRT-PCR assays according to the manufacturer’s protocol. Each reaction contained: 0.5 *μl* of forward primer (0.5 *μM*), 0.5 *μl* of reverse primer (0.5 *μM*), 5 *μl* of 5x qPCRmix-HS SYBR+Low-ROX, 0.5 *μl* of solution containing cDNA obtained using the commercial kit SuperScript III cDNA Synthesis CellDirect in accordance with the manufacturer’s recommendations, and 18.5 *μl* of DEPC-treated and autoclaved water. Lyophilized primers were previously diluted using DEPC-treated and autoclaved water.

### PCR cycles were run as follows:

2 *min* at 50*°C*, then denaturation for 10 *min* at 95*°C* followed by 40 cycles of 15 *s* at 95*°C*, and 1 *min* at 60*°C*. Fluorescence was measured following each annealing and extension phase. Melt curve was produced to confirm a single gene-specific peak and to detect primer/dimer formation by heating the samples from 70 to 95*°C* in 0.5*°C* increments with a dwell time of 10 seconds at each temperature while continuously monitoring the fluorescence. The reactions were set up in a 96-well PCR plate (Applied BioSystems, USA) in three technical replicates.

Controls included non-RT controls (Total RNA without reverse transcription was used to monitor genomic DNA contamination) and non-template controls (Water template).

For relative quantification (RQ) of examined genes, the Ct mean of three reference genes (Gapdh, H2afz and Rpl4) for normalization were used to minimize the effect of fluctuations in the expression level of each individual gene. To calculate the gene expression level (RQ) of target genes, the ΔCT-method was used as described (37).

### Time-lapse microscopy:

Time-lapse microscopy of oocytes was performed using an inverted microscope Biolam-P1, equipped with oblique lighting system and ProgRes CT video camera. This microscope was installed into CO_2_-incubator (5% CO_2_/95%air, 37*°C*). To simplify identification of different samples, oocytes were placed in specialized WoW (Well-of-the-well) culture dishes (PrimoVision, Vitrolife). The culture of oocytes was performed for 40 *hr* in α-MEM medium. For time-lapse microscopy, 31 group A oocytes, 45 group B oocytes and 21 group C oocytes, totally 97 oocytes were used.

Images were captured every 5 *min*. In order to minimize the effect of light exposure on cultured oocytes, RODOS-3 time relay (Olimp, Russia) was used to set up the following lighting regime: 1 *min* light on/4 *min* light off. During the light-on stage, images were made using ProgRes Capture Pro 2.9. XnView software (Pierre-Emmanuel Gougelet) and VirtualDub (Avery Lee) freeware applications were used for further processing of the obtained images and construction of time-lapse video files.

### Data analysis:

**Figure 2.**
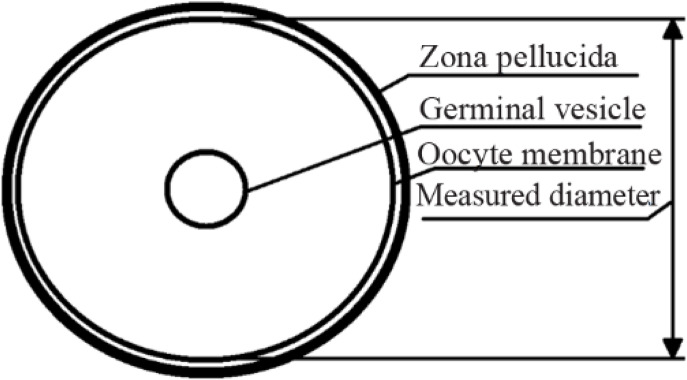
Scheme illustrating oocyte diameter measurement

Ovarian follicles and oocytes imaging was performed using Nikon Eclipse 2000TE equipped with Hoffman modulation contrast system. For the measurement of oocytes, freely available software ImageJ (NIH, USA) was used. The following approach was used to minimize potential measurement errors in oocyte diameter estimation: firstly, the total area of oocyte on focused image was measured, then, the diameter of oocyte was calculated by using the formula D=2(S/π)^1/2^. The inner diameter of oocyte excluding zona pellucida was calculated (Illustration is given in figure 2).

Statistical significance was calculated by the Kruskal-Wallis test using Statistica 7.0 software (Dell, Inc., USA). To verify the hypothesis of normal distribution, Shapiro-Wilk normality test was used (Test was also performed using Statistica 7.0 software). The Kruskal-Wallis test was applied because no normal distribution was observed in all cases and then multiple comparison of mean ranks for all groups was performed. To analyze the differences in the progression to different morphological stages of meiosis in oocytes from various groups 2x2 -contingency tables and chi-square test were used. Differences were considered statistically significant if p<0.05.

### Ethical committee approval:

All manipulations were performed according to the rules of MSU Biological Ethical committee recommendations.

## Results

**Figure 3.**
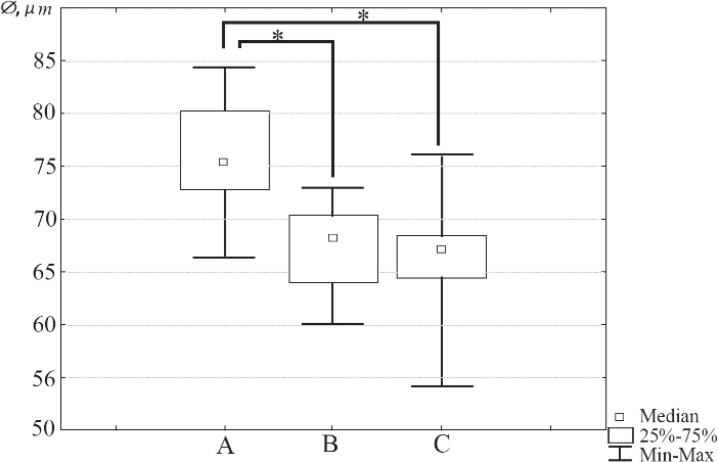
The box-and-whisker plot illustrating diameter of oocytes from three different groups: A–oocytes obtained from mature mice (3 months, oocyte n=71, Median=75.37 *μm*, IQR: 73.25–80.21), B–oocytes obtained from immature mice (3 weeks, n=85, Median=68.23 *μm*; Interquartile range: 64.04–70.05), C–oocytes isolated from ovarian follicles cultured for 8 days in alginate hydrogel (n=51, Median=67.07 *μm*, IQR: 64.42–68.47). Group B and group C oocytes are smaller than group A oocytes (p<0.01 in both cases, multiple comparisons of mean ranks), there is no difference between the diameters of group B and group C oocytes (p>0.9, multiple comparisons of mean ranks)

**Figure 4.**
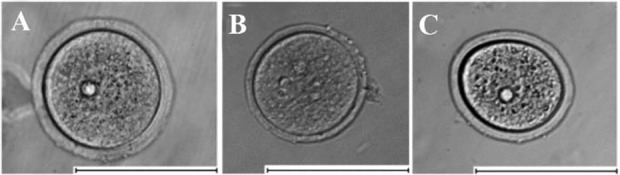
Photo of typical oocytes from three different groups: A–oocytes obtained from mature mice (3 months), B– oocytes obtained from immature mice (3 weeks), C–oocytes isolated from ovarian follicles cultured for 8 days in alginate hydrogel. The scale bar is 100 *μm*

The diameter of oocytes from three different groups was measured as described above. Totally, 207 oocytes were measured (Group A n=71, group B=85, group C=51). The statistical analysis has revealed that the median diameters of oocytes retrieved from immature mice (Median=68.23 μm; Interquartile range: 64.04–70.05) and oocytes isolated from ovarian follicles cultured in vitro (Median=67.07 *μm*, IQR: 64.42–68.47) differed significantly from the median diameter of fully-grown oocytes retrieved from mature mice (Median= 75.37 *μm*, IQR: 73.25–80.21, p<0.01) in both cases. There was no statistically significant difference between the diameters of oocytes retrieved from immature mice and isolated from ovarian follicles cultured *in vitro* (p>0.9). The box-and-whisker plot representing the values of oocyte diameter for three different oocyte groups is given in figure 3. Photo of typical oocytes retrieved from mature and immature mice and isolated from ovarian follicles cultured *in vitro* are shown in figure 4A–C.

To evaluate the meiosis progression in different groups of oocytes, the time-lapse microscopy was applied. Images of three different morphological stages corresponding to various stages of meiosis in oocyte were obtained using time-lapse microscopy: presence of GV (Germinal vesicle–corresponds to the arrest of oocyte at prophase I), GVBD (Germinal vesicle breakdown–corresponds to the transition from prophase I to further stages) and PBE (Polar body extrusion–corresponds to the metaphase II stage). It was found that oocytes retrieved from immature mice and oocytes obtained after ovarian follicles *in vitro* culture show significantly lower rates of progression to GVBD compared to the oocytes retrieved from mature mice (p=0.02 and p=0.01, correspondingly). Furthermore, progression to MII stage also was lower in oocytes from groups B and C compared to the oocytes retrieved from mature mice (p<0.01 and p<0.01, correspondingly) (Table 2). There was no significant differences in progression to MII or GVBD stages between B and C groups (p=0.26 and p=0.72 correspondingly). Moreover, oocytes retrieved from immature mice showed prolonged progression from GVBD to PBE stage and oocytes isolated from ovarian follicles cultured *in vitro* also exhibited delayed transition from GVBD to PBE stages (Table 3). Video illustrating the differences in meiotic progression of typical oocytes from three investigated groups is given in Supplementary Material.

**Table 2.** Morphological stages of oocytes from three different groups after 40 *hr* of culture

	**GV**	**GVBD**	**PBE**
**Group A (n=31)**	0.31 (0%)	2.31 (7%)	29.31 (93%)
**Group B (n=45)**	7.45 (16%)	24.45 (53%)	14.45 (31%)
**Group C (n=21)**	4.21 (19%)	8.21 (38%)	9.21 (43%)

A–oocytes obtained from mature mice (3 months), B–oocytes obtained from immature mice (3 weeks), C–oocytes isolated from ovarian follicles cultured for 8 days in alginate hydrogel. Group B and group C oocytes show significantly lower rates of progression to GVBD compared to group A oocytes (p=0.02 and p=0.01, correspondingly, chi-square test). Progression to MII stage was also lower in oocytes from groups B and C compared to group A oocytes (p<0.01 and p<0.01, correspondingly). There was no significant differences in progression to MII or GVBD stages between B and C groups (p=0.26 and p=0.72, correspondingly)

**Table 3.** Median time required for the transition to different morphological stages in oocytes from three different groups

	**GV->GVBD**	**GVBD->PBE**
**Group A**	85 *min* (n=31)	570 *min* (n=29)
**Group B**	90 *min* (n=38)	1020 *min* (n=14)
**Group C**	110 *min* (n=17)	1195 *min* (n=9)

(A–oocytes obtained from mature mice (3 months), B–oocytes obtained from immature mice (3 weeks), C–oocytes isolated from ovarian follicles cultured for 8 days in alginate hydrogel) during *in vitro* culture. No differences in GVBD time occurrence have been observed across all three groups (p=0.38, Kruskal-Wallis test). Significant differences in the time required for GVBD-MII transition have been observed between group A and B (p< 0.01, multiple comparisons of mean ranks), group A and C (p<0.01, multiple comparisons of mean ranks); no significant difference in the GVBD-MII transition time was detected across groups B and C (p>0.9, multiple comparisons of mean ranks)

To analyze gene expression levels in oocytes from three different groups, qRT-PCR analysis was performed. It was found that gene expression levels of Cdk1 and Ccnb (Encoding components of MPF-complex, CDK1 and Cyclin B1, respectively), and Wee2 (Which encodes regulator of MPF complex activity) are similar in three groups of oocytes (Obtained from mature and immature mice and isolated from ovarian follicles cultured *in vitro*, p=0.69, p=0.91 and p=0.29, correspondingly). The expression levels of Ccnh (Which encodes Cyclin H, a component of CAK-complex which regulates MPF-complex) and Epab (Encoding ePAB protein, responsible for the translation initiation) were significantly lower in oocytes isolated from ovarian follicles cultured *in vitro* in comparison to oocytes obtained from mature and immature mice (For Ccnh p=0.01 and p=0.03 correspondingly; for Epab p=0.02 in both cases). The expression levels of Mos (Which encodes regulator of MAPK-cascade activity) were significantly lower in oocytes obtained from immature mice as compared with oocytes obtained from mature mice (p<0.01). The box-and-whisker plots illustrating gene expression levels of the investigated genes are given in figure 5.1–6.

**Figure 5.**
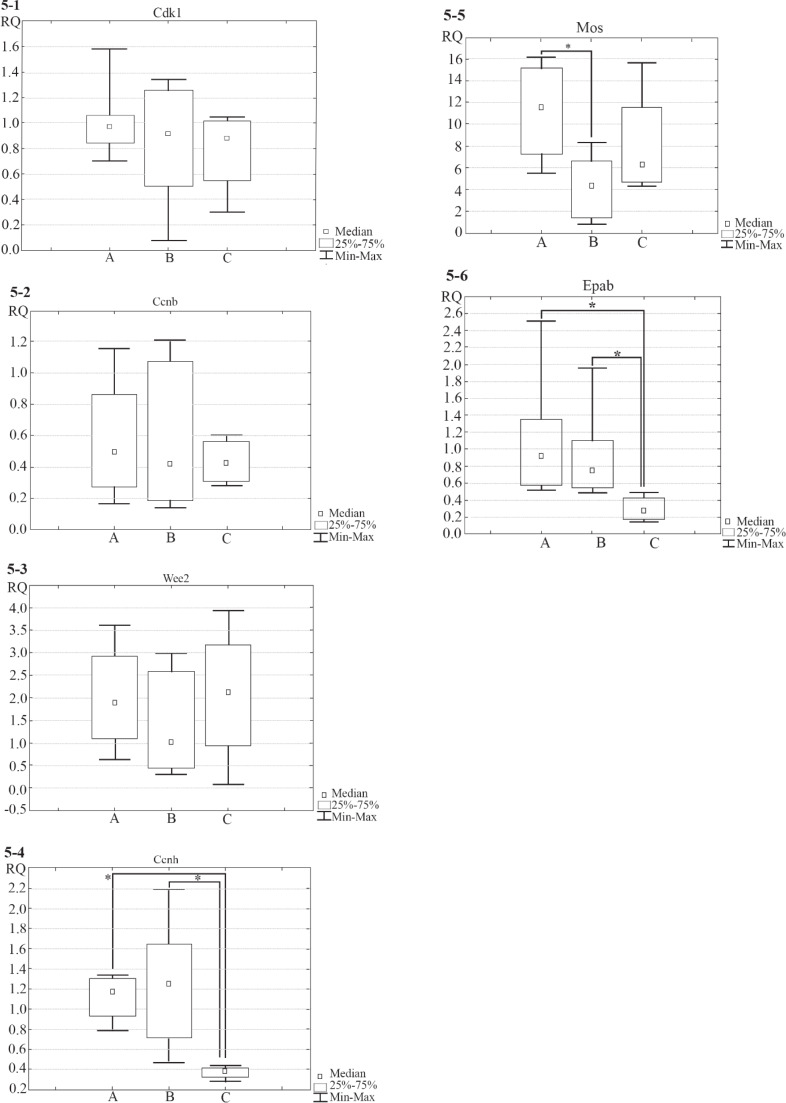
The box-and-whisker plot illustrating gene expression levels (Cdk1, Ccnb, Wee2, Ccnh, Mos and Epab– Figures 5.1–5.6, correspondingly) in oocytes from three different groups: A–oocytes obtained from mature mice (3 months, samples n=8), B–oocytes obtained from immature mice (3 weeks, n=8), C–oocytes isolated from ovarian follicles cultured for 8 days in alginate hydrogel (n=6). Figure 5.1-Cdk1: group A (Median= 0.96, IQR 0.83–1.06), group B (Median=0.90, IQR 0.51–1.25), group C (Median=0.88, IQR 0.55–1.01), no differences were found in the expression levels across three groups (p=0.69, Kruskal-Wallis test); Figure 5.2-Ccnb: group A (Median= 0,50, IQR 0.31–0.86), group B (Median=0,42, IQR 0.18–1.07), group C (Median= 0,42, IQR 0.31–0.56), no differences were found in the expression levels across three groups (p=0.91); Figure 5.3-Wee2: group A (Median=1.88, IQR 1.11–2.94), group B (Median= 0.94, IQR 0.46–2.58), group C (Median=2.12, IQR 0.95–3.17), no differences were found in the expression levels across three groups (p=0.29); Figure 5.4-Ccnh: group A (Median=1.17, IQR 0.93–1.30), group B (Median=1.26, IQR 0.64–1.65), group C (Median=0.38, IQR 0.32–0.41), statistically significant differences were found between groups A and C, B and C (p=0.01 and p=0.03, correspondingly, multiple comparisons of mean ranks), no differences were found in the expression levels between groups A and B (p>0.9, multiple comparisons of mean ranks); Figure 5.5-Mos: group A (Median=11.52, IQR 7.29–15.10), group B (Median=4.29, IQR 1.38–6.55), group C (Median= 6.24, IQR 4.69–11.57), statistically significant differences were found between groups A and B (p<0.01, multiple comparisons of mean ranks), no differences were found in the expression levels between groups A and C (p=0.89, multiple comparisons of mean ranks), B and C (p=0.51, multiple comparisons of mean ranks); Figure 5.6-Epab: group A (Median= 0.92, IQR 0.58–1.35), group B (Median=0.75, IQR 0.54–1.10), group C (Median=0.27, IQR 0.17–0.41), statistically significant differences were found between groups A and C, B and C (p=0.02 in both cases, multiple comparisons of mean ranks), no differences were found in the expression levels between groups A and B (p>0.9, multiple comparisons of mean ranks)

## Discussion

In the current work, GV stage oocytes were used because in GV oocytes maternal mRNAs are bound by MSY2 protein which prevents their destruction. Once oocyte meiotic resumption is induced, phosphorylation of MSY2 occurs, resulting in maternal mRNAs degradation (38). Therefore, the use of GV oocytes allowed us to obtain more accurate data about the expression levels of investigated genes in oocytes. If GVBD or MII oocytes are used for qRT-PCR analysis, the examined oocytes could differ in time since the GV breakdown occurred resulting in higher variability in the amount of stable mRNAs, thus leading to the distortion of qRT-PCR results.

In this research, C57BL/6J inbred mice were used. Traditionally, in studies dedicated to the investigation of ovarian follicles, *in vitro* culture techniques from F_1_ hybrid (2, 10) or outbred (3–6) mice are used. In this work, expression levels of several key genes which are responsible for meiosis in mouse oocytes were evaluated. The use of outbred mice in such study seems to be unsuitable due to the potential high genetic variance across the individuals used in the experiments. F_1_ hybrids often have more uniform phenotype than inbred animals (39), that is potentially due to the buffering effects of heterozygosity (40–41). However, it has been shown that in F_1_ hybrids individual variation in gene expression patterns may be higher in comparison to parental inbred line (42). To avoid any potential fluctuations which may affect gene expression levels, this research work was done in inbred mice. The C57BL/6J strain is highly available and commonly used in reproductive biology (43–46).

In the current work, multicomponent culture mediums were used based on the α- MEM for the ovarian follicle growth and maturation as they are commonly used in similar works (4, 13, 18, 47). According to the data presented in literature, several special supplements were added to the culture medium. FSH (Follicle-stimulating hormone), insulin-transferrin-selenium, and serum were used because these additives support folliculogenesis (4, 13, 18, 47). L-carnitine was added because it stimulates lipid metabolism thus increasing meiotic competence in oocytes retrieved from ovarian follicles cultured *in vitro* (2). Ascorbic acid was added into S-medium and P-medium; it has been demonstrated that addition of ascorbic acid into the culture medium improves remodeling of extracellular matrix in ovarian follicles cultured *in vitro*, thus promoting their growth and development (48). To prevent bacterial growth, gentamicin was also added into the culture medium.

During the culture period, half of the medium was replaced with P-medium every two days. P-medium contained double amounts of additives in comparison to the S-medium. Most of the applied substances have a short half-life (49–50); therefore, the concentration of many additive components on the second day of culture should be too low. Since only half of medium was changed, the P-medium was used to make the concentration of additives the same as the one at the beginning of culture procedure. During the preliminary experiments, it was found that the increase in serum concentration in culture medium from 5% to 10% does not lead to changes in growth of follicles encapsulated in the alginate.

**Figure 6.**
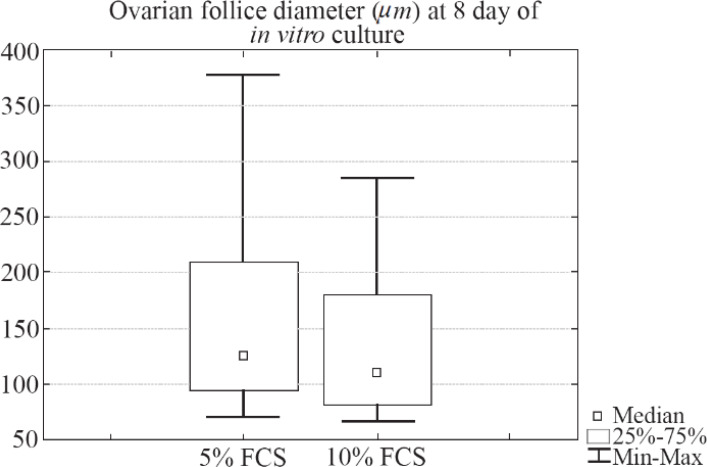
The box-and-whisker plot illustrating diameter (*μm*) of ovarian follicles cultured in 5% FCS medium (Cultured follicles n=51) *vs*. 10% FCS (Cultured follicles n=28) medium on day 8. No statistically significant differences between these two groups were observed (p=0.23, Mann-Whitney U-test). 5% FCS: Median=125.27, IQR 96.20–209.31; 10% FCS: Median=110.11, IQR 82.54–180.23

Briefly, individual secondary ovarian follicles retrieved from 2-week-old mice were cultured in two different types of culture medium. The first type of culture medium was composed of α-MEM with addition of FSH (0.1 *IU/ml*), FCS (5%), insulin (5 *μg/ml*), transferrin (5 *μg/ml*), selenium (5 *ng/ml*), and gentamycin (80 *μg/ml*). The second type of culture medium was composed of α-MEM with addition of FSH (0.1 *IU/ml*), FCS (10%), insulin (5 *μg/ml*), transferrin (5 *μg/ml*), selenium (5 *ng/ml*), and gentamycin (80 *μg/ml*). The whole volume of medium was replaced every two days. No statistically significant differences between these two groups were observed (p=0.23, Mann-Whitney U-test) and the box-and-whisker plot illustrating diameter (*μm*) of ovarian follicles cultured in two different types of culture medium (5% *vs*. 10% FCS) on day 8 of culture is given in figure 6 (In 5% FCS medium, Median=125.27, IQR 96.20–209.31; 10% FCS: Median=110.11, IQR 82.54–180.23).

In this work, only half volume of culture medium was changed each time to avoid potential drying of alginate beads and to simplify the culture medium replacement. When only the half of the medium is changed, it is simple to control the location of alginate bead and prevent its catching into the pipette tip.

The M-medium used for the imitation of ovulation *in vitro* contained human chorionic gonadotropin (hCG) and epidermal growth factor (EGF), two commonly used inducers of oocyte maturation (11, 13–15, 47). Ascorbic acid was not supplemented into the M-medium because its’ antioxidative properties can prevent elevation of ROS (Reactive oxygen species) in culture medium and ovarian follicles while ROS is required for induction of oocyte maturation during ovulation (17); thus, addition of ascorbic acid could suppress ovulation processes *in vitro*.

Pimobendan is another component added into the M-Medium. Pimobendan is a selective blocker of PDE3A (Phosphodiesterase 3 A type) (51). When PDE3A in oocyte is inactivated, PKA (Protein kinase A) produces cAMP which, in turn, prevents GVBD (17). PDE3A is expressed in oocyte but not in the cumulus cells (52), therefore the action of its blockers could not lead to the distortion of molecular cascades in the follicular cells and normal ovulation processes should occur. Thus, addition of PDE3A results in ovulation of meiotically immature GV oocyte.

In preliminary experiments, it was revealed that oocytes obtained from ovarian follicles cultured *in vitro* in alginate hydrogel have lower diameter in comparison to fully-grown oocytes obtained from mature mice. Also, these oocytes showed reduced developmental competence. To analyze the potential reasons of low competence of oocytes cultured *in vitro*, it was decided to investigate the status of oocytes from three groups: fully grown oocytes retrieved from mature 3-month-old mice (Group A, control group “gold standard”, ∼75 *μm* in diameter), oocytes retrieved from immature 3-week-old mice (Group B, ∼68 *μm*) and oocytes obtained from ovarian follicles cultured *in vitro* in alginate hydrogel (Ovarian follicles were isolated from 2-week-old mice, group C, ∼67 *μm*). Oocytes retrieved from immature 3-week-old mice were used for two reasons. First, diameter of oocytes retrieved from immature mice is very close to the diameter of oocytes obtained from ovarian follicles cultured *in vitro* in alginate. Second, the duration of ovarian follicle *in vitro* culture is 8 days. Ovarian follicles for *in vitro* culture were isolated from 2-week-old mice, and therefore the development time of oocytes cultured in vitro in alginate hydrogel corresponds well to the development time of oocytes isolated from 3-week-old mice.

Oocytes retrieved from ovarian follicles cultured *in vitro* showed significantly lower rate of first polar body extrusion compared to oocytes retrieved from mature mice (Table 2). Moreover, for these oocytes, the time required for GVBD and first polar body extrusion was also significantly longer in comparison to oocytes retrieved from both mature and immature mice (Table 3).

The efficiency of ovarian follicle culture was significantly lower in the current work as compared to the similar works (4, 8, 13). The potential reason of such discrepancy may be the fact that in the current work, different mouse strains were used which may be more sensible to the *in vitro* culture conditions. In the preliminary studies conducted in F_1_ hybrid mice, higher efficacy of ovarian follicle was revealed in *in vitro* culture procedures (Data not shown).

Gene expression levels of Cdk1, Ccnb and Wee2 were similar in three groups of oocytes (Obtained from mature and immature mice and isolated from ovarian follicles cultured *in vitro*), indicating that the basic mechanisms of MPF-complex regulation at transcriptional level work properly in oocytes from three different groups. Also, gene expression levels in oocytes isolated from ovarian follicles cultured *in vitro* differ from that levels in oocytes obtained from both mature and immature mice. Thus, expression levels of Ccnh and Epab were significantly lower in oocytes isolated from ovarian follicles cultured *in vitro* in comparison to the oocytes retrieved from mature and immature mice.

Ccnh gene encodes protein Cyclin H which is a catalytic subunit of CAK-complex. The key function of CAK-complex is activation of MPF-complex via phosphorylation (17). Thus, decrease in Ccnh expression can cause attenuated GVBD in oocytes isolated from ovarian follicles cultured *in vitro* in comparison to the normal GVBD in oocytes retrieved from mature and immature mice. The CAK-complex is a moonlighting protein complex (53–55). It has been shown that CAK-complex in addition to its function as a regulator of MPF-complex is also a component of the general transcription factor TFIIH (55–56). Moreover, it has been demonstrated that in some cell types, CAK-complex can also regulate gene expression by direct phosphorylation of transcription factors, including the retinoic acid receptor and peroxisome proliferator-activated receptor γ (57–58). Therefore, the lack of Cyclin H which is a component of CAK-complex in oocyte may lead to the serious malfunction of biochemical cascades.

Epab gene is responsible for synthesis of ePAB (Embryonic poly(A)-binding) protein. This protein is specific for oocytes; it binds to the poly-A terminal sites of mRNA and triggers their translation (26–28). The reduced levels of Epab transcripts can lead to reduction of ePAB levels in oocyte. Decrease in the concentration of proteins that initiate translation can be critical, since it causes decrease in the production of different important proteins. Reduction of ePAB production in oocyte can result in the impairment of meiosis; moreover, this may lead to the delayed embryo development after fertilization of such oocyte. It is because during initial stages of embryo cleavage, maternal proteins and mRNAs are used. Therefore, decrease in Epab expression may be the reason of delayed meiosis in oocytes isolated from ovarian follicles cultured *in vitro* in comparison to meiotic progression in oocytes retrieved from mature and immature mice.

Interestingly, differences in the expression levels of Mos gene were found in oocytes isolated from mature and immature mice. MOS protein is an important regulator of meiotic maturation which controls MAPK-pathway. MAPK-pathway has multiple functions in oocyte meiosis. Although in case of mouse oocytes, activation of MAPK-pathway is not essential for GVBD (59), stimulation of MAPK-pathway leads to GVBD (22–23); MAPK-pathway is also responsible for normal spindle assembly in oocytes (24). Moreover, MAPK cascade regulates maternal mRNA translation in oocytes (25). Thus, a decrease in expression of Mos can lead to the reduction in percentage of oocytes displaying normal meiosis obtained from immature mice in comparison to the oocytes retrieved from mature mice.

Therefore, though oocytes obtained from immature mice and obtained after *in vitro* culture of ovarian follicles are of similar size and they demonstrate impaired meiosis, the causes of abnormal oocyte development appear to be different in these two groups.

## Conclusion

In the current work, it was revealed that oocytes obtained from immature mice or after ovarian follicle *in vitro* culture have significantly lower diameter in comparison to oocytes obtained from mature mice (68, 67 and 75 *μm*, correspondingly). Moreover, oocytes retrieved from immature mice or from ovarian follicles cultured *in vitro* show impaired meiosis progression and demonstrate aberrations in gene expression levels: Ccnh and Epab (For oocytes obtained from ovarian follicles cultured *in vitro*), and Mos (For oocytes obtained from immature mice).

Meiosis in oocyte is a very complex process orchestrated by multiple molecular pathways and regulated at different levels: transcriptional, translational and by post-translational modifications. In this study, mRNA expression levels of only six genes regulating meiosis in oocytes were examined, thus the data presented in the current article is just a tip of the iceberg and the reasons of impaired meiosis in oocytes may be due to impaired translation or transcription of other key genes responsible for meiosis regulation such as Pabpc1, Pabpc2, Msy2 and Patl2 (Regulation of translation), Aurka, Aurkb and Aurkc (Spindle organization), securin and separase (Chromosome segregation).

The *in vitro* culture system of ovarian follicles used in this work is very similar to commonly used systems in this area (4, 8, 12–13, 16, 18, 47). However, this kind of system is far from that to be highly physiological and able to reproduce exact conditions *in vivo*. The cyclic change of the culture medium and absence of ovarian stroma elements in the culture system may negatively influence the quality of retrieved oocytes (60–61). Ovarian follicle *in vitro* culture is an actively developing research area and novel approaches are proposed to make the *in vitro* culture more effective: non-contact co-culture of a large number of follicles (3), co-culture of follicles in conditioned medium (62–63), culture of ovarian follicles in conditions mimicking different rigidity properties of ovarian cortex and medulla (14, 16, 64–65).

The development of ovarian follicle *in vitro* culture systems is required to make the conditions of *in vitro* culture closer to those observed *in vivo*, *i.e*. to make reconstruction of ovary *in vitro* possible. And the novel data obtained in the current work will allow performing adequate analysis of oocyte status in further researches dedicated to the ovarian follicle *in vitro* culture.
